# Integration of SNP Disease Association, eQTL, and Enrichment Analyses to Identify Risk SNPs and Susceptibility Genes in Chronic Obstructive Pulmonary Disease

**DOI:** 10.1155/2020/3854196

**Published:** 2020-12-29

**Authors:** Yang Liu, Kun Huang, Yahui Wang, Erqiang Hu, Benliang Wei, Zhaona Song, Yuqing Zou, Luanfeng Ge, Lina Chen, Wan Li

**Affiliations:** ^1^College of Bioinformatics Science and Technology, Harbin Medical University, Harbin, China; ^2^Department of Respiratory, The Second Affiliated Hospital, Harbin Medical University, Harbin, China

## Abstract

Chronic obstructive pulmonary disease (COPD) is a complex disease caused by the disturbance of genetic and environmental factors. Single-nucleotide polymorphisms (SNPs) play a vital role in the genetic dissection of complex diseases. In-depth analysis of SNP-related information could recognize disease-associated biomarkers and further uncover the genetic mechanism of complex diseases. Risk-related variants might act on the disease by affecting gene expression and gene function. Through integrating SNP disease association study and expression quantitative trait loci (eQTL) analysis, as well as functional enrichment of containing known causal genes, four risk SNPs and four corresponding susceptibility genes were identified utilizing next-generation sequencing (NGS) data of COPD. Of the four risk SNPs, one could be found in the SNPedia database that stored disease-related SNPs and has been linked to a disease in the literature. Four genes showed significant differences from the perspective of normal/disease or variant/nonvariant samples, as well as the high performance of sample classification. It is speculated that the four susceptibility genes could be used as biomarkers of COPD. Furthermore, three of our susceptibility genes have been confirmed in the literature to be associated with COPD. Among them, two genes had an impact on the significance of expression correlation of known causal genes they interact with, respectively. Overall, this research may present novel insights into the diagnosis and pathogenesis of COPD and susceptibility gene identification of other complex diseases.

## 1. Introduction

Chronic obstructive pulmonary disease (COPD) is an inflammatory disease of the respiratory system, which is one of the most important causes of death in most countries [1]. A myriad of evidence has demonstrated that genetic factors are associated with the development and aggravation of COPD [2]. To date, there are no ideal therapies that retard the progression of disease or mortality [3]. The in-depth study on the genetic mechanism of COPD and the screening of related biomarkers could provide an important theoretical basis for the treatment and prevention of COPD.

Usually, SNPs could be used as genetic markers to explore the genetic mechanism of complex diseases [4]. In recent years, great progress has been made in SNP research on COPD-related diseases. Deng et al. demonstrated that the SNP rs8004738 of *SERPINA1* gene was linked to a high risk of COPD by SNP genotyping [5]. A polymorphism rs12068264 in the cathepsin S gene was identified as associated with susceptibility to COPD in the Chinese Han population [6]. Based on luciferase assay, real-time polymerase chain reaction (real-time PCR) and other methods, Li et al. unraveled that the upstream functional SNP rs12654778 of the *ADRB2* gene specifically affected the expression of *ADRB2* in COPD [7]. Notably, these studies were performed for an individual or some specific known genes or SNPs. In hereditary complex diseases, SNP disease-associated test method can detect the potential genetic association between genetic variation on the genome and specific diseases or traits and has emerged as an effective way to facilitate COPD research [8]. Using disease association analysis, numerous SNPs identified on loci associated with lung function and COPD from different studies showed a high consistency [9]. The genetic variation rs7937 in COPD mined by association study might influence the DNA methylation and expression levels of corresponding genes in the blood, which was associated with the progression of COPD [10]. The SNP disease association study was able to identify a large number of COPD-related risk sites, providing a basis for further screening of disease mechanism research and clinical application but failed to reflect the expression correlation of their products. Expression quantitative trait loci (eQTLs) have taken gene expression as a trait to explore the correlation between genetic variation and gene expression and effectively served as a biomarker for COPD research [11]. The eQTL analysis elucidated that SNP rs793391 in the *SUMF1* gene was related to increased risk of COPD [12]. Sun et al. integrated protein quantitative trait loci (pQTLs) identified by meta-analysis with eQTLs reported in previous studies to facilitate the discovery of pathogenesis of COPD [13]. Based on the association study combined with the eQTL analysis, new disease-associated biomarkers could be found. Four lung eQTLs were uncovered on 4q31, 4q22, and 19q13 of susceptible loci using Nguyen et al.'s association results [14]. Combining genome-wide association study with lung eQTL analysis, several disease-related SNPs were found to regulate the lung mRNA expression levels of new asthma genes [15]. These genetic analyses based on eQTL usually build a single mathematical model, and the datasets of eQTL and disease association analysis were derived from different cohorts. The correlation between the results need to be further verified.

Nowadays, the next-generation sequencing (NGS) data can provide SNP-related information and transcript expression information to explore the occurrence and development of diseases from the genomic level and transcription level [16]. It is a strong data support for the screening of COPD-related biomarkers. Mayhew et al. studied COPD subtypes based on high-throughput sequencing data of the 16S ribosomal RNA gene [17]. Combining the miRNA expression profile obtained by NGS and qPCR, the pathogenesis of miR-10a-5p and miR-146a-5p in asthma and COPD was revealed [18]. Expression profiles of mRNA and miRNA in bronchial epithelial cells generated by miRNA and transcriptome sequencing confirmed that the PI3K-Akt signaling pathway played a crucial role in COPD [19]. COPD candidate gene *CHRNA5* and risk variant rs8040868 were mined by exon sequencing of lung development-related genes [20]. However, up to now, there is no relevant report on the identification of COPD-related biomarkers by RNA-seq data.

**Figure 1:**
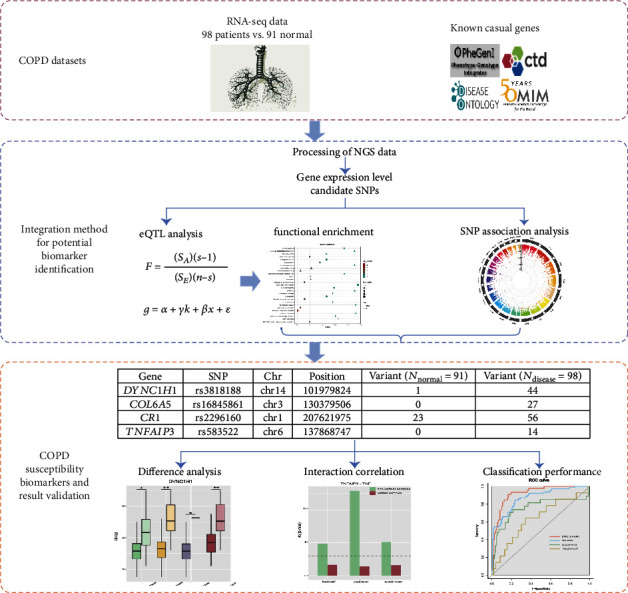
Overall study design. Abbreviations: COPD: chronic obstructive pulmonary disease; NGS: next-generation sequencing; eQTL: expression quantitative trait loci. Linear regression and ANOVA are two models applied in eQTL analysis.

Herein, a new integration strategy was proposed using RNA-seq data of 98 COPD patients and 91 normal samples. The susceptibility genes and risk SNPs of COPD were identified from the perspectives of SNP sequence variation and expression quantitative trait locus (Figure 1). It is expected to increase the understanding of the pathogenesis of COPD at the genetic level, provide new biomarkers for the diagnosis and treatment of COPD, and shine a light for studying the intrinsic molecular mechanism of other complex diseases.

## 2. Materials and Methods

### 2.1. Data Source

COPD-related RNA-seq data in raw SRA format compiled from 189 samples (accession number: GSE57148) was downloaded from the Gene Expression Omnibus (GEO) (https://www.ncbi.nlm.nih.gov/geo/) database, including 98 COPD patients and 91 normal lung tissue samples.

Human reference genome sequence in FASTA format and GTF format data for gene annotation (version number: release 38.89) were procured from the ENSEMBL (http://asia.ensembl.org/index.html) database. The GTF data involves the position information of the functional elements of protein-coding genes and other nonprotein-coding genes such as lncRNA and miRNA on chromosomes. A VCF format file encompassing known single-nucleotide polymorphisms, insertions, and deletion sites on the human reference genome was gleaned from ENSEMBL.

We searched the Online Mendelian Inheritance in Man (OMIM) (https://omim.org/downloads/), the Comparative Toxicogenomics Database (CTD) (http://ctdbase.org/), the Phenotype-Genotype Integrator (PheGenI) (https://www.ncbi.nlm.nih.gov/gap/phegeni), and the Disease Ontology (DO) (http://disease-ontology.org/) databases to obtain 32 genes that had been described in the databases as being involved in COPD.

### 2.2. Processing of NGS Raw Data

The SRA format data of 189 samples was transformed into a paired-end sequencing FASTQ format file using the SRA Toolkit (https://www.ncbi.nlm.nih.gov/sra/docs/toolkitsoft/) and the transformed file containing base information and corresponding sequencing quality information. The paired-end reads were aligned to the human reference genome using the multisequence alignment software HISAT2, and the reordering, deduplication, quality control, and SNP calling of the alignment results were performed using SAMtools (http://samtools.sourceforge.net) and BCFtools (https://samtools.github.io/bcftools/bcftools.html). SNPs with a mapping quality score less than 30 were removed to reduce noise and to control the quality. SNPs identified by different sequencing depths were analyzed to determine the depth that would stabilize the number of SNP mutations, and the Hardy-Weinberg equilibrium test was executed to obtain candidate SNPs. The COPD-related SNPs were mapped to functional elements of the corresponding genes using SnpEff v4.0 (http://snpeff.sourceforge.net/index.html) based on the position information of the SNP.

For a given SNP, the genotype that is in the human reference genome sequence is considered the reference against which all other forms are compared. This reference genotype is called the wild-type genotype, while others are variant genotypes. In our manuscript, variant samples were samples with variant SNP genotypes, and nonvariant samples were samples with wild-type SNP genotypes.

Transcript splicing and quantification of transcripts and genes were carried out using StringTie v1.3.3 (https://ccb.jhu.edu/software/stringtie/index.shtml) combined with HISAT2 alignment results and gene-annotated GTF files. Genes with FPKM values greater than 0.5 in at least 30% of the samples were included in downstream analysis.

### 2.3. Integration Method for Identification of Potential Biomarkers

To identify potential biomarkers of COPD, the SNP association analysis was performed based on genotype data derived from variant calling results. Concurrently, the correlation analysis between genetic mutation and gene expression was also carried out. The results of these two steps were integrated after exploring the biological functions of the relevant genes. The detailed process is as follows.

### 2.4. SNP Association Study for COPD

Association between variants and disease phenotype (disease/normal data) was evaluated based on genotypes of candidate SNPs, and the genetic variants most likely to affect traits were selected by *p* value. PLINK, a powerful analysis toolset containing many functional modules for SNP disease association study or other genetic studies [21] was used. Using PLINK v1.90 beta (http://www.cog-genomics.org/plink2/) software, all SNPs identified in the cohort were involved in the association study, and the significant *p* value of the Chi-square test of each SNP was calculated. Level of 0.001 was considered the significant threshold of Hardy-Weinberg equilibrium (HWE), and the standard of 0.1 was set as minor allele frequency (MAF). SNPs that did not meet the thresholds for HWE and MAF were filtered out.

### 2.5. eQTL Analysis

For each SNP, we adopted two computational algorithms for eQTL analysis. (1) For each risk SNP, according to its mutation, the COPD samples and the normal samples were divided into three mutant sample groups: nonmutation, homozygous mutation, and heterozygous mutation. To investigate the differences in the expression levels of variant SNPs between normal and disease samples as well as variant and wild-type SNP genotypes in disease samples, the analysis process was implemented by employing the ANOVA:

(1) $$\begin{array}{c} \text{F}=\frac{\left({\text{S}}_{\text{A}}\right)\left(\text{s}‐1\right)}{\left({\text{S}}_{\text{E}}\right)\left(\text{n}-\text{s}\right)}, \end{array}$$

where *s* is the number of groups divided based on mutation; *n* is the total number of samples; *S*_*A*_ is the efficacy sum of squares between mutation types, that is, it is the between-group variation caused by mutation; and *S*_*E*_ is the error sum of squares, also known as within-group variation. The calculated *p* value (FDR < 0.05) was to measure statistical significance.

(2) In addition, we proposed a linear regression model that simulated genotypes as additive linear effects to analyze the association between the genotype and gene expression. The eQTL analysis was performed by calculating the relationship of the transcript-SNP, that is, survey SNP was correlated with gene expression. Here, we assumed that the association between gene expression and genotype is linear:

(2) $$\begin{array}{c} g=\alpha +\gamma k+\beta x+\varepsilon , \end{array}$$

where *x* is the genotype, *g* is the value of gene expression, *k* is the covariate of disease phenotype, *α* is the intercept, *β* is the slope coefficient, *γ* is the weight of covariate, and *ε* indicates deviation. The Student *t*-test for analyzing gene-SNP association was executed, and statistical significance after FDR was required (FDR < 1 × 10^−6^).

The SNPs identified by both the above algorithms were termed as candidate risk SNPs, and the corresponding mapped genes were defined as COPD candidate genes.

### 2.6. Enrichment Analysis

The clusterProfiler R package is presented within the Bioconductor project and automated the process of biological-term classification and the enrichment analysis of gene clusters [22]. Here, gene classification and enrichment analysis of combining COPD candidate genes with known causal genes were performed based on the annotation package http://org.Hs.eg.db [23]. The statistically significant (FDR adjusted *p* value < 0.05) functional category containing both candidate genes and known causal genes were treated as a disease-related functional category.

Whereafter, we regarded COPD candidate genes that were significantly enriched in disease-related functional categories and also mapped to significant COPD-related SNPs in an association study as disease susceptibility genes, and the corresponding candidate risk SNPs were defined as disease risk SNPs.

## 3. Results

### 3.1. Candidate SNPs

SNPs identified by different sequencing depths (6, 7, 8, 9, and 10) were analyzed to determine the depth that would stabilize the number of SNPs. There were no significant differences in the distribution of variant sites obtained for different sequencing depths after sequencing depth reached 10. Moreover, It was suggested that only >10× coverage was required to ensure 89% accuracy and 92% sensitivity for single-nucleotide variations [24, 25]. Therefore, the subsequent analysis was conducted on SNPs with a sequencing depth of 10 for SNP calling. VCF format files were merged using VCFtools, and the Hardy-Weinberg equilibrium test was further performed. As a result, 16,357 candidate SNPs were identified after setting a significance threshold of 0.05.

### 3.2. COPD-Related SNPs

**Figure 2:**
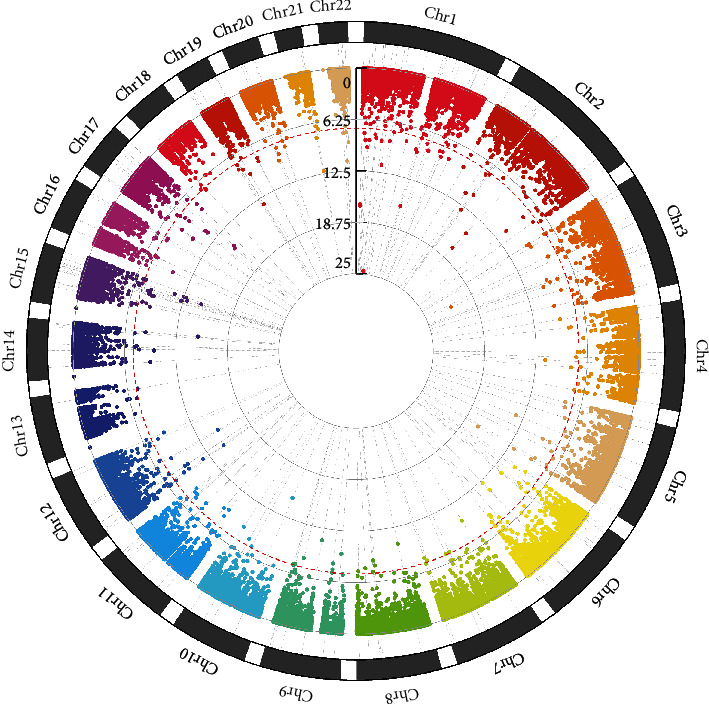
Circle Manhattan plot summarizing SNPs associated with COPD. The *x*-axis corresponds to 22 autosomal chromosomes and different colors to distinguish different chromosomes. The *y*-axis shows the -log10 (*p* value). The red dashed line depicts the threshold for significance (*p* value < 5 × 10^−8^).

SNP disease association study was implemented on SNPs with a sequencing depth of 10 using PLINK, revealing the significant differences between disease-affected individuals versus normal individuals. Here, a total of 190 COPD-related candidate risk SNPs achieved the 5 × 10^−08^ level of significance (Table 1), and their chromosomal positions on each autosome are represented in Figure 2.

### 3.3. Candidate Genes of COPD

Using the ANOVA algorithm, 558 SNPs with significant differences (FDR < 0.05) related to expression level were mined for variant SNPs in disease and normal samples, which were annotated to 428 genes. For variant and wild-type SNP genotypes in COPD samples 1,162 SNPs were detected (FDR < 0.05) to be correlated with the expression level in these samples, and 737 genes were annotated. After merging, 1,428 SNPs and their corresponding 895 genes linked to expression were finally singled out.

850 SNPs in 561 genes were associated with expression level in all samples using linear regression models (FDR < 1 × 10^−6^). Combining the results of the above two methods, a total of 198 COPD candidate genes with 318 candidate risk SNPs involved in the expression were mined.

### 3.4. COPD Susceptibility Genes

**Figure 3:**
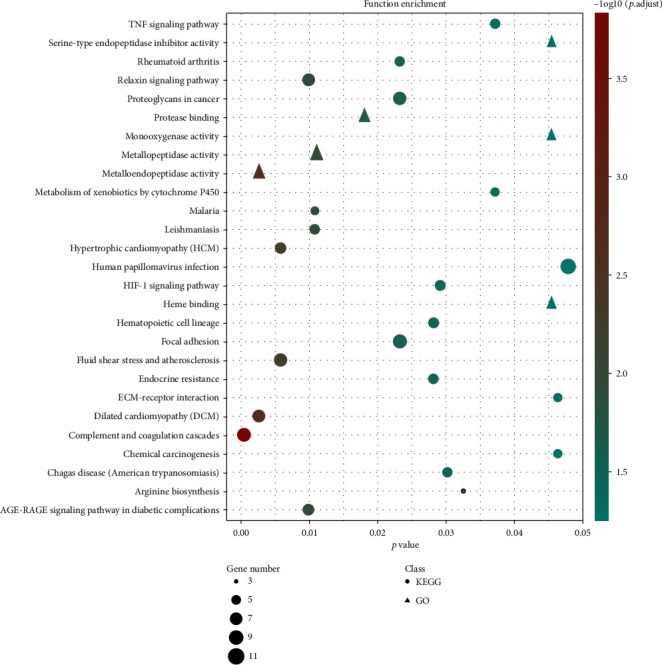
Enriched biological functions for the disease. Triangle and circle dots correspond to the Gene Ontology (GO) functional category and Kyoto Encyclopedia of Genes and Genomes (KEGG) pathway class, respectively. The size of the dot indicates the number of candidate susceptibility genes enriched in the biological function. The redder the point, the higher the significance of the biological function.

Kyoto Encyclopedia of Genes and Genomes (KEGG) and Gene Ontology (GO) functional enrichment analyses were performed for the candidate genes and known causal genes using R software, and the statistically significant threshold of 0.05 was set. The two sets of genes were significantly enriched in 21 KEGG pathways and 6 functional categories, including protease binding and monooxygenase activity (Figure 3). These processes were verified to be related to COPD in the literature [26, 27]. Combining 38 candidate genes that synergized with known causal genes in the disease-related functional categories with COPD-related SNPs in the association results, our method finally recognized 4 risk SNPs and 4 susceptibility genes (Table 2).

### 3.5. The Validation of Susceptibility Genes

#### 3.5.1. COPD Susceptibility Genes Differences

Differential expression analysis was implemented to investigate the differences of the four susceptibility genes for the following situations: (i) between normal and disease samples, (ii) between variant and nonvariant samples, (iii) between variant and nonvariant normal samples, and (iv) between variant and nonvariant disease samples. Significantly different genes between normal and disease samples indicated their relationship to the disease. Significantly different genes between variant and nonvariant (all/disease/normal) samples indicated the change between variant and wild-type SNP genotypes.

**Figure 4:**
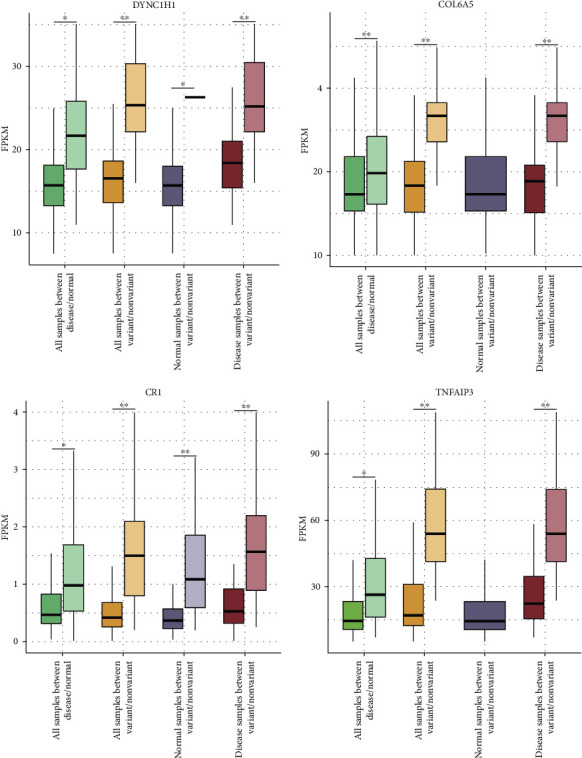
Boxplot of expression levels of four susceptibility genes. Four groups of samples were used for differential analysis in each gene (i.e., *DYNC1H1*, *COL6A5*, *CR1*, and *TNFAIP3*), and the boxplot with similar colors indicates that each sample type is internally divided into two groups. The significant difference is measured according to *p* value (^∗∗^ < 10^−5^, ^∗^10^−5^ − 0.05).

For each group, differential expression analysis was executed using the R-package limma with core function lmFit (*p* < 0.05) (Figure 4). It turned out that the four susceptibility genes were significantly different not only between normal and disease samples (*p* < 0.001) but also between variant and nonvariant samples as well as variant and nonvariant disease samples (*p* < 1 × 10^−5^). Of note, there was also a significant trend for *DYNC1H1* and *CR1* between the variant and nonvariant normal samples. However, *COL6A5* and *TNFAIP3* variants were absent in normal samples.

#### 3.5.2. Correlation between COPD Susceptibility Genes and Known Causal Genes

**Figure 5:**
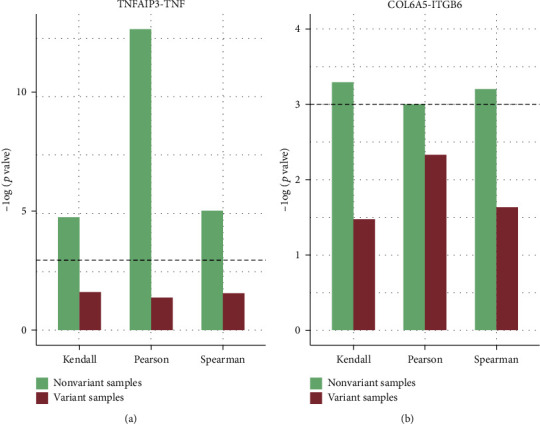
Correlation of genes. (a) Histogram of coexpression analysis of *TNFAIP3* and *TNF*. (b) Histogram of coexpression analysis of *COL6A5* and *ITGB6*. The dotted line represents the threshold level of 0.05. Three function methods were used, respectively, namely, Kendall, Pearson, and Spearman rank. Label along the *y*-axis indicates the significance of the extent of correlation. The green histogram refers to the nonvariant samples, while the red histogram is the variant samples.

The functional properties of proteins are usually clarified by protein interactions in protein-protein interaction (PPI) networks [28]. Therefore, *TNFAIP3* and *TNF*, *COL6A5*, and *ITGB6* were mined as known causal genes interacting with susceptibility genes from the String (https://string-db.org/), menthe (http://mentha.uniroma2.it/), and MINT (https://mint.bio.uniroma2.it/) databases. Coexpression analysis of each COPD susceptibility gene and its interactive known causal gene was performed for variant and nonvariant samples using the Kendall, Pearson, and Spearman methods, respectively (Figure 5). The results exhibited that there was no significant correlation between each pair in the variant samples while a significant correlation in the nonvariant samples. In other words, the extent of the correlation of each pair showed a dramatic change between the variant and nonvariant samples. It implied that the variant SNPs in COPD susceptibility genes were likely to have an underlying impact on the correlation between their corresponding genes and the interactive known causal ones.

#### 3.5.3. Classification Performance of COPD Susceptibility Genes

**Figure 6:**
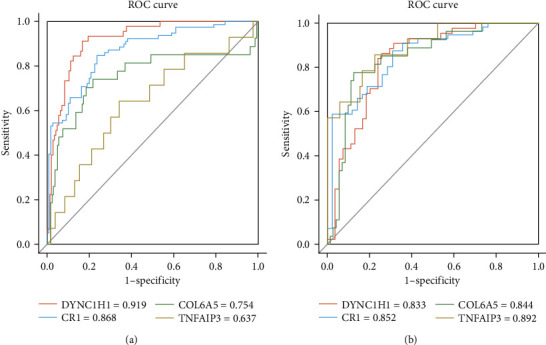
The ROC curve of four susceptibility genes. (a) ROC curve of all samples for variant/nonvariant classification. (b) ROC curve of disease samples for variant/nonvariant classification. Sensitivity is the true positive rate (TPR), and specificity is the true negative rate (TNR).

To further verify the validity of susceptibility genes as potential biomarkers for COPD, a classifier was constructed using an SVM model with a linear kernel [29], characterized by FPKM values of each COPD susceptibility gene. We evaluated the leave-one-out cross-validation performance using an average ROC area under the curve (AUC) metrics, which is a common way to overcome the overfitting problem. In classifying variant/nonvariant samples, three of the four susceptibility genes have an AUC value of more than 0.75 (Figure 6). Besides, the AUC value of each susceptibility gene classifying variant/nonvariant disease samples was above 0.83, indicating that their classification performances were excellent. The vast majority of genetic variation occurred in disease samples. Therefore, the polymorphisms of the four susceptibility genes showed significant expression differences in the disease status. They also reflect the consistency between the variation and expression level.

## 4. Discussion

Chronic obstructive pulmonary disease is chronic bronchitis or emphysema characterized by airflow obstruction that can further develop into a common chronic disease of pulmonary heart disease. Based on RNA-seq data for COPD, we proposed a strategy for identifying disease risk SNPs and susceptibility genes. SNP disease association study was performed on candidate SNP genotype information with a sequencing depth of 10. Additionally, eQTL analysis of these candidate SNPs was implemented using two algorithms to detect COPD candidate genes. The COPD susceptibility genes and their corresponding risk SNPs were finally obtained by functional enrichment analysis containing known causal genes.

We selected sequencing depth with stable identification of SNP mutation sites to conduct SNP calling. No significant differences were detected in the distribution of variant sites obtained for different sequencing depths. Therefore, genotype information of SNP with a sequencing depth of 10 could be used as a reliable basis for our subsequent research, which was also suggested by other researchers. Furthermore, we examined our risk SNPs in the SNPedia database that stored disease-associated SNPs. One of our four risk SNPs, rs2296160 located on *CR1*, could be found to be associated with COPD or lung function in the database.

In the eQTL analysis, the overlapping genes of two eQTL algorithms were considered accurate candidate genes. One algorithm was to analyze the variance of three mutation forms for corresponding sample types to obtain candidate eQTL genes. The other was a simple linear regression model constructed on putting the genotype as the additive linear effects to calculate the transcript-SNP relationship and picked out *cis*-eQTL genes.

Moreover, susceptibility genes for COPD were identified by integrating SNP disease association, eQTLs, and the results of enrichment analysis. Among them, the obtained COPD candidate genes accounted for 22% and 35% of two eQTL analysis algorithms, respectively. These genes showed the correlation between SNP and gene expression from multiple perspectives. Thus, they are reliable and effective for exhibiting strong disease susceptibility both in the functions and in the extent of SNP mutation. The susceptibility genes and known causal genes were enriched together in many disease-related functional categories. In the function of protease binding, the multifunctional glycoprotein osteopontin, which was highly upregulated in the airways of patients with COPD, protected the bacteria by binding to the bacterial surface, resulting in OPN reducing lysozyme-induced death of streptococcus pneumoniae [30]. As for hematopoietic cell lineage pathway, analysis of whole-genome lncRNA expression in the lung tissue of COPD smokers indicated that smoking was associated with activation of metabolic pathways, whereas COPD transcripts were related to hematopoietic lineages, intermediate metabolism, and immune system processes [31]. Similarly, the focal adhesion pathway was closely related to COPD. Bufei Yishen Formulation (BYF) has been shown to have a short-term therapeutic effect in COPD rats. Due to oxidoreductase-antioxidant activity, focal adhesions, tight junctions, or lipid metabolism, genes regulated in COPD and lung tissue of BYF-treated rats were discerned in relevant omics analysis [32].

The four susceptibility genes were significantly different between disease/normal samples or variant/nonvariant disease samples, especially in the latter which suggested more significant differences. The extent of expression correlation of our susceptibility genes and the known causal genes had varied dramatically between the variant and nonvariant samples. One risk SNP (rs2296160) and three susceptibility genes were confirmed to be linked to the disease in the literature. SNP rs2296160 was observed to be statistically associated with increased risk of idiopathic pulmonary fibrosis [33]. The susceptibility gene *TNFAIP3* was an endogenous negative regulator of the transcription factor *κ*B (NF-*κ*B) signaling, and NF-*κ*B was central to the pathogenesis of many inflammatory diseases, such as COPD [34]. Validation of the susceptibility gene *CR1* unveiled that cigarette smoke might prevent COPD-related cardiovascular disease [35]. *COL6A5* gene was a novel collagen VI gene identified at a single locus on human chromosome 3q22.1, and its mRNA expression was restricted to a few tissues such as the lung [36].

**Figure 7:**
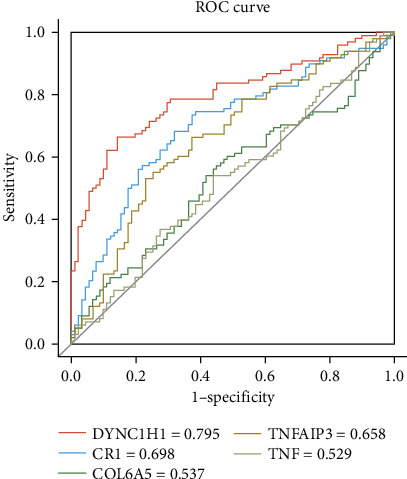
The ROC curve of four susceptibility genes and *TNF*. The objects of the binary classification are all samples classified by disease and normal status. The gray line represents the ROC curve of the known causal gene *TNF*.

In addition, for all samples according to disease and normal classification, known causal gene *TNF* acted synergistically on a functional category with susceptibility genes *COL6A5*, *CR1*, and *TNFAIP3*, respectively.  Figure 7 showed that the AUC value of *TNF* was 0.529, which was lower than the classification performance of four susceptibility genes between disease and normal samples. Meanwhile, compared with the classification performance of 12 known causal genes in the same functional category, our susceptibility genes were higher than most known causal genes. The classification performance of four susceptibility genes for variant/nonvariant disease samples was better than that for disease/normal samples because the samples with variation were almost derived from disease samples. Taken together, the four genes could effectively serve as susceptibility genes for COPD.

There are several limitations to our study. Firstly, a certain amount of noise exists in RNA-seq data, which might have a slight impact on our results. Secondly, it is very difficult for just several genes or SNPs to capture all the features due to the complexity of COPD. Machine learning has been launched to predict disease outcomes using distinct types of data including images and clinical records. Thus, combining other types of data with large amounts of genomics or omics data for the advanced analysis to answer clinical questions is the direction of future studies.

## 5. Conclusions

In this study, based on the high-throughput sequencing data of COPD, we proposed an integrated strategy to identify risk SNPs and susceptibility genes from the perspectives of expression-related SNPs and SNP association. Besides, functional characteristics, interaction correlation, classification performance, and differences between groups were considered to evaluate the accuracy and robustness of our methods and results. Especially, among our results, three genes were associated with COPD in the literature and one risk SNPs was characterized as related to COPD in the SNPedia database. Conclusively, COPD susceptibility genes identified by our method are credible and may be taken as biomarkers of disease. Considering that COPD is a chronic complex disease that can develop into respiratory failure, SARS-CoV-2, which is currently raging around the world, is also a pulmonary disease that mainly causes severe lung damage to infected patients. With the in-depth research and the acquisition of large-scale data, the risk SNPs and susceptibility genes identified based on our method using the related SARS-CoV-2 data might provide directions to the research investigations on the prevention and treatment strategies of the disease. In addition, our framework might be also applied to identify susceptibility genes of other genetically related complex diseases, such as cardiovascular diseases and diabetes.

## Figures and Tables

**Table 1:** Part of significant SNPs from SNP disease association study.

Chr	SNP	Position	Ref	Alt	*p* value
1	snv14	630211	C	T	1.789e − 17
1	rs9283154	633714	A	G	3.193e − 17
1	rs172933	7784620	T	C	1.272e − 08
1	rs2295079	11262508	C	G	1.938e − 09
1	rs1571982	16625221	A	T	2.667e − 10
1	rs3738097	21568323	T	C	1.998e − 09
1	rs11263839	35929124	A	C	1.035e − 08
1	rs2937378	36305205	G	C	2.273e − 25
1	rs2275188	39283249	G	A	4.364e − 09
1	rs1056438	53214206	T	C	2.219e − 09

**Table 2:** Susceptibility genes and risk SNPs.

Gene	SNP	Chr	Position	Variant (*N*_normal_ = 91)	Variant (*N*_disease_ = 98)
*DYNC1H1*	rs3818188	chr14	101979824	1	44
*COL6A5*	rs16845861	chr3	130379506	0	27
*CR1*	rs2296160	chr1	207621975	23	56
*TNFAIP3*	rs583522	chr6	137868747	0	14

^∗^ *N*_normal_ is the number of normal samples. *N*_disease_ the number of disease samples. Variant represents the number of variants in the sample type.

## Data Availability

The datasets generated during and analyzed during the current study are available in the GEO repository, https://www.ncbi.nlm.nih.gov/geo/query/acc.cgi?acc=GSE57148.
